# Determination of Saponin Content in Hang Maidong and Chuan Maidong via HPLC-ELSD Analysis

**DOI:** 10.1155/2016/7214607

**Published:** 2016-01-27

**Authors:** Xian-En Li, Yu-Xia Wang, Peng Sun, Deng-Qun Liao

**Affiliations:** Institute of Medicinal Plant Development, Chinese Academy of Medical Sciences and Peking Union Medical College, Beijing 100193, China

## Abstract

Zhejiang and Sichuan are regarded as two genuine producing areas of Ophiopogonis radix in China. To study the difference in the quality of Ophiopogonis radix from these two places, the contents of three reported bioactive saponins, that is, ophiopogonins B, D, and D′, in tubers and fibrous roots of* Ophiopogon japonicus* from Cixi city of Zhejiang and Santai county of Sichuan were quantified using HPLC-ELSD method and compared. Ophiopogonin B and ophiopogonin D′ content in tubers of HMD were higher than those in radix of CMD, whereas ophiopogonin D in HMD was about twice lower than that of CMD. Three ophiopogonins were all detected in fibrous roots of both HMD and CMD. Their averaged content in fibrous roots of CMD was about twice higher than that in tubers of CMD. Ophiopogonin D in fibrous roots of CMD was about five times higher than that of HMD. Our biochemical assay revealed that fibrous roots and tubers of CMD can be of an important saponin source, especially for ophiopogonin D.

## 1. Introduction

*Ophiopogon japonicus* (Thunb) Ker-Gawl. (also called maidong in Chinese) belongs to the family Liliaceae. Ophiopogonis radices, dried tubers of maidong, are routinely used in traditional Chinese medicine to nourish lung and body fluids, benefit the stomach, and remove stresses [1, 2]. Maidong is widely grown in several provinces of China including Sichuan, Zhejiang, Hubei, Anhui, Yunnan, and Jiangsu [3, 4]. However, it is mainly distributed in Zhejiang province and Sichuan province, which is separately called Hang maidong (HMD) and Chuan maidong (CMD). Chinese traditional medical knowledge (TMK) has shown that therapeutic effects of maidong produced in Zhejiang (mainly Cixi city) and Sichuan (mainly Santai county), especially HMD, are superior to those cultivated in other places. Aqueous extract of HMD and CMD tubers showed the different pharmacological effects on rats [5]. With the economic prosperity, industry development, and change of agricultural layout in China, cultivation and the yield of HMD, like many other crops, have been greatly decreased in Zhejiang province. Sichuan is now becoming the primary maidong-producing place, followed by Hubei province [4]. So it is of importance to study the difference between Hang maidong and Chuan maidong in their chemical constituents, which provides the information on the quality control for future cultivation of maidong in new places and breeding new varieties containing desired or higher amount of bioactive compounds.

A lot of chemical compositions have been isolated from radix or fibrous roots of* Ophiopogon japonicus*, including steroidal saponins, polysaccharides, homoisoflavonoids, and amino acids [1, 2, 6–9]. Steroidal saponins and homoisoflavonoids are considered as the main bioactive pharmacological compounds in Ophiopogonis radix and thus extensively studied. Homoisoflavonoid profiling and quantitative assay showed that the content and compositions of specific homoisoflavonoids differed in tubers of CMD and HMD [10]. HMD contained more homoisoflavonoids such as methylophiopogonanones A and B than CMD. Lin et al. [11] found that CMD had a higher amount of total saponins composed of diosgenin and ruscogenin than HMD. Ophiopogonins B, D, and D′ were three bioactive saponin compounds [12–15]. Except that ophiopogonin D was assayed simultaneously in HMD and CMD, showing the different content between these two places [16], ophiopogonins B and D′ have not been measured simultaneously in both HMD and CMD. In this study, we established an HPLC-ELSD method to measure and compare these three saponin compounds of Ophiopogonis radix and fibrous roots that were harvested in Zhejiang province and Sichuan province of China.

## 2. Materials and Methods

### 2.1. Plant Material

Maidong tubers and fibrous roots were collected from Santai county, Sichuan province, and Cixi city, Zhejiang province of China (Table 1). The samples were washed, deenzymed at 105°C for 15 min, and baked to dryness at 60°C. They were then ground and passed through a 40-mesh-size. The powders were stored in the desiccator till the analysis.

### 2.2. Extraction

Two grams of powder was extracted under reflux with 100 mL methanol for 1 hour and filtered with cheesecloth. The extract was evaporated to dryness in a rotary evaporator at 65°C under the reduced pressure and redissolved in 40 mL of water. The obtained solution was subsequently extracted with 20 mL of petroleum ether. The petroleum ether phase was discarded, and the fraction was then extracted three times with 90 mL of water-saturated butanol. The pooled butanol portions were evaporated to dryness at 65°C under the reduced pressure and dissolved in 2 mL methanol for the HPLC-ELSD analysis below.

### 2.3. Determination of Steroidal Saponins

HPLC grade acetonitrile was purchased from JT Baker Inc. (USA). Standards of ophiopogonin B (≥98%) and ophiopogonin D′ (≥98%) were obtained from Sichuan Weikeqi Biotechnology Co., Ltd., China. The standard ophiopogonin D (≥98%) was purchased from Shanghai Yilin Biotechnology Co., Ltd., China. The concentrations of ophiopogonins B, D, and D′ in the mixed stock standard solution were, separately, 0.082 mg, 0.111 mg, and 0.095 mg in 1 mL of methanol. 2, 4, 6, 8, 10, 15, and 20 *μ*L of the standard mixture were separately taken and diluted in methanol for HPLC-ELSD analysis to make their corresponding calibration curves. Calibration curves were constructed by plotting the logarithm of the HPLC-ELSD peak areas versus the logarithm concentration of each standard.

Ten microliters of extracts and standards were run at 25°C on a Waters 600E system equipped with an Agela Venusil ASB C18 column (4.6 mm × 150 mm, 5 *μ*m), Waters 2424 evaporative light-scattering detector, and 2707 autosampler (USA). A linear gradient mobile phase containing acetonitrile (A) and water (B) was applied as follows: 45% A to 55% A for 30 min, 55% A to 45% A for 5 min, and 45% A held for another 5 min. The flow rate was 1.0 mL/min. The other ELSD settings included the following: drift tube temperature, 70°C; nitrogen flow rate, 2.4 mL/min; and heating power level of the sprayer, 60%. Three replicates of each sample were measured.

### 2.4. Soil Sampling and Analysis

Soil samples were taken from the top 20 cm layer in maidong-growing fields of Zhejiang and Sichuan provinces. Five soil samples from the same field were collected according to the “S” shape arrangement and then pooled as one representative sample of the field. After being air-dried, the 20-mesh-size soil was prepared for determining soil properties including pH value, electrical conductivity (EC), available P and K, total N, and organic matter content, which were assayed by the central laboratory of Institute of Plant Nutrition and Resources, Beijing Academy of Agriculture and Forestry Sciences, Beijing. Soil pH was measured in 1 : 2.5 soil (gram)/water (mL) solution by a pH meter. Soil electric conductivity was measured in a 1 : 5 soil/water extract using a conductivity meter. Available P and K, total N, and organic matter content were determined by molybdenum antimony blue colorimetry, flame photometry, Kjeldahl nitrogen method, and dichromate oxidation, respectively. The standard procedures for each method were followed during the extraction and quantification.

## 3. Results and Discussion

### 3.1. Validation of HPLC-ELSD Methodology

**Figure 1:**
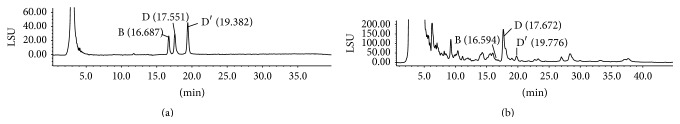
HPLC-ELSD chromatographs of standard mixture (a) and maidong extract (b).

Saponins have no UV absorption due to the absence of chromophores in their structures. Also, their UV absorption at *λ* 200–210 nm was generally affected by polysaccharides and interfering peaks, making UV readings unstable and irreproducible. HPLC-ELSD method has successfully determined the content of ophiopogonins D and D′ in maidong [17]. So we adopted this method to quantify ophiopogonins B, D, and D′ of maidong.  Figure 1 showed that our HPLC conditions can separate ophiopogonins B, D, and D′ from other peaks. Standards of ophiopogonins B, D, and D′ were eluted separately at 16.687 min, 17.551 min, and 19.382 min. Their corresponding compounds in the sample extract were detected at 16.594 min, 17.672 min, and 19.776 min, respectively.

The linear regression equations obtained were ophiopogonin B, *y* = 1.022*x* + 12.96, *R* ^2^ = 0.9991; ophiopogonin D, *y* = 1.032*x* + 13.47, *R* ^2^ = 0.9995; and ophiopogonin D′, *y* = 1.398*x* + 13.12, *R* ^2^ = 0.9986. For the precision estimation, a sample extract was assayed successively for 6 times. The precisions calculated for ophiopogonin B, ophiopogonin D, and ophiopogonin D′ were 0.91%, 0.59%, and 0.87%, respectively. This indicated that our instrument was in good precision condition. Repeatability was obtained via parallel preparation of six sample extracts from the same batch. RSD for ophiopogonin B, ophiopogonin D, and ophiopogonin D′ were 1.00%, 1.32%, and 1.11%, respectively, which indicated that our HPLC method was reproducible.

To check the stability of extracts, the same sample extract was assayed separately at 0, 2, 4, 8, 12, and 24 h after extraction. The values of relative standard deviation (RSD) for ophiopogonin B, ophiopogonin D, and ophiopogonin D′ were 1.22%, 1.52%, and 1.31%, respectively, indicating that our sample extract remained stable at least till 24 hours.

To test the sample recovery, the standard solutions with known concentrations were added to 2 grams of maidong powder at the beginning of extraction. The recovery rates for standards of ophiopogonin B, ophiopogonin D, and ophiopogonin D′ were 98.20%, 103.80%, and 99.30%, respectively.

### 3.2. Content of Three Saponins in Tubers of Hang Maidong and Chuan Maidong

The content of three steroidal saponins in tubers of HMD and CMD was shown in Table 1. Overall, ophiopogonins B, D, and D′ were very low in tubers of maidong, regardless of their origin places. The total amount of three saponins in different places of HMD was from 82.8 *μ*g/g to 139.04 *μ*g/g, with an average of 114.22 *μ*g/g, and, in CMD, from 70.16 *μ*g/g to 132.05 *μ*g/g, with an average of 107.71 *μ*g/g. However, the amount of individual saponins between HMD and CMD was significantly different. The content of ophiopogonins B and D′ in tubers of HMD was significantly higher than that in CMD. For example, ophiopogonin B in tubers of HMD was between 26.25 *μ*g/g and 57.03 *μ*g/g, with an average of 41.90 *μ*g/g; however, the content of ophiopogonin B in CMD was only from 7.10 *μ*g/g to 27.7 *μ*g/g, with an average of 13.98 *μ*g/g. The content of ophiopogonin D in tubers of CMD was about twice higher than that in HMD, which was consistent with Yu et al.'s result [16].

### 3.3. Content of Saponins in Fibrous Roots of Hang Maidong and Chuan Maidong

Our HPLC-ELSD analysis revealed that fibrous roots of both CMD and HMD contained ophiopogonins B, D, and D′ (Table 2). The total content of these three saponins in fibrous roots of CMD ranged from 181.76 *μ*g/g to 368.65 *μ*g/g, with an average of 241.06 *μ*g/g. The total amount of three saponins in fibrous roots of HMD varied from 59.75 *μ*g/g to 143.46 *μ*g/g, with an average of 114.50 *μ*g/g. Wu et al. found that the total saponin content in fibrous roots of CMD was higher than that in tubers of HMD and CMD [18]. So was the case with these three ophiopogonins. Their total content in fibrous roots of CMD was about twice higher than that in tubers of HMD and CMD, due to the high amount of ophiopogonin D in CMD. Only ophiopogonin D was found significantly higher in fibrous roots of CMD than in those of HMD. Ophiopogonins B and D′ were lower in fibrous roots of CMD than in HMD. HMD had a similar amount of ophiopogonins in its tubers and fibrous roots (Tables 1 and 2).

The difference in saponin content between HMD and CMD, together with other bioactive compounds such as homoisoflavonoids and polysaccharides, was determined by a combination of cultivation habits, environmental conditions, and genetic difference between HMD and CMD. For example, Chuan maidong has to be harvested yearly, due to high temperatures and rainfall in the summer which make most tubers turn black and decay [19, 20]; Hang maidong can grow well all year round and is usually harvested three years after plantation when HMD has its maximum yield [21] and higher amounts of metabolites including homoisoflavonoids and polysaccharides [19, 20]. Our soil chemical characterization showed that, after two or three years' plantation, HMD-growing soil contained lower available P and total N than CMD-growing fields (Table 3). Generally, HMD-growing soil pH was higher than CMD-growing soil, which was consistent with Zhang et al.'s previous results [22]. EC value, available K, and organic matter content varied between the fields of both provinces and showed no significant difference between HMD- and CMD-growing places. Literatures document that HMD and CMD are genetically differential [23]. When CMD and HMD were grown in the same place, it was found that ecological conditions in Cixi city, Zhejiang province, were optimal for the growth and quality (tuber size) of Hang maidong compared to CMD [24]. However, no such parallel experiments have been conducted to reveal how greatly environmental factors and the genetic difference of HMD and CMD influence the accumulation of secondary metabolites in them. This will be one of important research aspects in future to understand ecological and genetic mechanisms underlying the metabolite difference between HMD and CMD, thus providing the important information on cultivating maidong in non-geoherbalism producing places and breeding new varieties.

## 4. Conclusion

Ophiopogonins B, D, and D′ in tubers and fibrous roots of* Ophiopogon japonicus* from Zhejiang and Sichuan were quantified and compared. Ophiopogonin B and ophiopogonin D′ content in tubers of HMD were higher than those in radix of CMD, whereas ophiopogonin D in HMD was about twice lower than that of CMD. Three ophiopogonins were all detected in fibrous roots of both HMD and CMD. Ophiopogonin D in fibrous roots of CMD was much higher than that of HMD. Our biochemical assay revealed that fibrous roots and tubers of CMD can be of an important saponin source, especially for ophiopogonin D. Our results also provided the information on the quality control for future cultivation of maidong in new places and breeding new varieties containing desired or higher amount of bioactive compounds.

## Figures and Tables

**Table 1:** The content of three saponins in tubers of HMD and CMD (*μ*g/g).

Origin^#^	Ophiopogonin B	Ophiopogonin D	Ophiopogonin D′	Total
Guangming	10.97 ± 2.23	75.29 ± 2.95	13.89 ± 6.29	100.15
Laoma	21.28 ± 2.72	82.87 ± 2.10	24.64 ± 9.67	128.79
Licheng	10.67 ± 1.70	94.43 ± 2.52	14.25 ± 9.94	119.35
Zhengsheng	8.64 ± 1.82	68.51 ± 4.57	15.73 ± 9.29	92.88
Xinde	7.10 ± 2.84	91.98 ± 2.03	18.56 ± 12.25	117.64
Lingxin	13.10 ± 1.13	84.61 ± 3.41	16.83 ± 4.40	114.54
Liuying	11.14 ± 2.63	64.38 ± 2.73	18.26 ± 11.99	93.78
Luxi	15.24 ± 3.21	36.33 ± 2.58	18.59 ± 12.39	70.16
Yongming	27.71 ± 3.72	90.03 ± 4.66	14.31 ± 8.12	132.05
**Mean (CMD)**	13.98 ± 6.60	76.49 ± 18.29	17.23 ± 3.36	107.71

Xiaolin	42.41 ± 3.97	59.40 ± 5.08	37.23 ± 6.74	139.04
Xingxing	57.03 ± 7.85	40.38 ± 0.27	23.42 ± 5.97	120.83
Qinchunbao	26.25 ± 0.74	23.66 ± 0.31	22.89 ± 1.71	82.8
**Mean (HMD)**	41.90 ± 15.40^**∗∗**^	41.15 ± 17.88^**∗**^	27.85 ± 8.13^**∗∗**^	114.22

^#^Town name, Santai county, Sichuan province; farm name and company name (3rd), Cixi city, Zhejiang province.

^*∗*^ *P* < 0.05  and ^*∗∗*^ *P* < 0.01  mean significant difference in the content between HMD and CMD, calculated by SPSS.

**Table 2:** The content of three saponins in fibrous roots of HMD and CMD (*μ*g/g).

Origin^#^	Ophiopogonin B	Ophiopogonin D	Ophiopogonin D′	Total
Guangming	15.42 ± 0.18	147.57 ± 0.87	26.90 ± 1.23	189.89
Laoma	12.08 ± 1.01	172.97 ± 4.10	90.01 ± 6.95	275.06
Licheng	14.96 ± 1.61	136.78 ± 2.59	30.02 ± 5.79	181.76
Zhengsheng	41.48 ± 5.11	144.56 ± 7.11	68.05 ± 8.77	254.09
Xinde	11.88 ± 2.12	171.69 ± 4.83	28.72 ± 10.98	212.29
Lingxin	16.04 ± 1.79	146.29 ± 2.85	31.65 ± 7.70	193.98
Liuying	31.34 ± 1.34	168.27 ± 4.95	69.51 ± 3.48	269.12
Luxi	27.28 ± 1.45	167.37 ± 4.55	30.08 ± 3.63	224.73
Yongming	65.94 ± 4.34	208.91 ± 4.82	93.8 ± 6.81	368.65
**Mean (CMD)**	26.27 ± 17.99	162.71 ± 22.00	52.08 ± 28.08	241.06

Xiaolin	20.99 ± 4.25	15.27 ± 1.64	23.49 ± 10.82	59.75
Xingxing	21.97 ± 3.08	79.71 ± 3.33	41.78 ± 8.25	143.46
Qinchunbao	54.92 ± 2.53	21.26 ± 0.55	64.12 ± 3.43	140.30
**Mean (HMD)**	32.63 ± 19.31	38.75 ± 35.60^**∗∗**^	43.13 ± 20.35	114.50

^#^Town name, Santai county, Sichuan province; farm name and company name (3rd), Cixi city, Zhejiang province.

^*∗∗*^ *P* < 0.01 means significant difference in the content between HMD and CMD, calculated by SPSS.

**Table 3:** Soil characteristics in HMD- and CMD-growing places.

Origin^#^	Available P (mg·kg^−1^)	pH value	EC (*μ*s cm^−1^)	Available K (mg·kg^−1^)	Organic matter (%)	Total N (%)
Guangming	53.25	6.87	158.90	68.51	2.06	0.09
Huayuan	92.52	5.27	126.00	59.76	1.70	0.10
Laoma	47.59	5.25	127.20	143.49	1.95	0.12
Laoma	118.19	5.26	122.20	113.50	1.77	0.13
Licheng	104.60	6.04	148.20	78.51	1.89	0.12
Licheng	47.77	6.30	165.50	93.50	1.66	0.12
Zhengsheng	44.75	6.43	259.00	168.48	1.83	0.12
Zhengsheng	47.59	6.57	162.10	261.32	1.75	0.12
Xinde	37.01	6.64	122.30	101.00	2.57	0.15
Xinde	22.47	6.81	91.80	61.01	1.29	0.11
Lingxin	46.26	6.92	138.90	73.51	1.60	0.10
Lingxin	53.06	6.91	134.20	101.00	1.55	0.11
Luxi	45.32	6.97	140.00	58.51	1.54	0.13
Yongming	111.02	6.85	276.00	180.98	2.12	0.13
**Mean (CMD)**	62.24 ± 30.50	6.36 ± 0.65	155.16 ± 51.40	111.65 ± 58.51	1.81 ± 0.31	0.12 ± 0.02

Xiaolin^1^	46.07	7.24	406.00	292.56	1.77	0.11
Xiaolin^2^	24.74	7.26	113.20	173.48	0.96	0.08
Xingxing^1^	26.07	7.63	110.90	87.25	1.52	0.07
Xingxing^2^	16.63	7.01	236.00	88.50	4.41	0.13
**Mean (HMD)**	28.38 ± 12.51^**∗**^	7.29 ± 0.26^**∗∗**^	216.53 ± 139.18	160.45 ± 96.88	2.17 ± 1.53	0.10 ± 0.03^**∗**^

^#^Town name, Santai county, Sichuan province; farm name, Cixi city, Zhejiang province. ^1^Two years after plantation. ^2^Three years after plantation.

^*∗*^ *P* < 0.05 and ^*∗∗*^ *P* < 0.01 mean significant difference in the content between HMD and CMD, calculated by SPSS.
